# Effects of Ischemic Preconditioning on Sport-Specific Performance in Highly Trained Taekwondo Athletes

**DOI:** 10.3390/sports12070179

**Published:** 2024-06-26

**Authors:** Maicon Rodrigues Albuquerque, Cristiano Arruda Gomes Flôr, Amanda Isadora Santos Ribeiro, Paulo Henrique Caldeira Mesquita, Emerson Franchini, Gilberto Candido Laurentino

**Affiliations:** 1 Combat Sports Laboratory, Department of Sports, Universidade Federal de Minas Gerais, Avenida Presidente Carlos Luz, 4664 Pampulha, Belo Horizonte CEP 31120-901, Minas Gerais, Brazil; amandoratkd@yahoo.com.br; 2 Centro de Treinamento Esportivo—CTE, Universidade Federal de Minas Gerais, Avenida Presidente Carlos Luz, 4664 Pampulha, Belo Horizonte CEP 31120-901, Minas Gerais, Brazil; 3 Centro Universitário Estácio BH, Rua Erê, 207—Prado, Belo Horizonte 30410-450, Minas Gerais, Brazil; cris.arruda77@gmail.com; 4 Aging and Metabolism Research Program, Oklahoma Medical Research Foundation, Oklahoma City, OK 73104, USA; paulo-mesquita@omrf.org; 5 Martial Arts and Combat Sports Research Group, Sport Department, School of Physical Education and Sport, University of São Paulo, Avenida Professor Mello Moraes, 65 Cidade Universitária, São Paulo 05508-030, São Paulo, Brazil; efranchini@usp.br; 6 Physical Activity Science Program, School of Arts, Science and Humanities, University of São Paulo, Rua Arlindo Béttio, 1000, São Paulo 03828-000, São Paulo, Brazil; gilbertolaurentino@usp.br

**Keywords:** combat sports, endurance performance, anaerobic performance, countermovement jump

## Abstract

Ischemic preconditioning (IPC), which involves episodes of blood flow restriction followed by reperfusion, has uncertain effects on athletes. Additionally, employing sports-specific tests that are highly familiar to athletes can enhance methodological rigor in determining IPC’s effects on taekwondo performance. This study aimed to investigate IPC’s influence on taekwondo athletes’ performance through two studies. To induce occlusion in both studies, the cuff was inflated to an individualized occlusion pressure established for each athlete’s lower limb, with four cycles of occlusion lasting five minutes each, alternated with five-minute reperfusion intervals. Both traditional frequentist statistics and Bayesian analysis were employed. In the first study, eleven high-level athletes were subjected to either IPC or a placebo (SHAM) procedure on both legs, followed by performing countermovement jumps (CMJs) and a specific taekwondo endurance test. However, no significant differences were observed in taekwondo endurance performance or CMJ between the IPC and SHAM conditions. The second study involved fourteen elite athletes who underwent the same IPC or SHAM conditions, performing CMJ and three bouts of the Multiple Frequency Speed of Kick test (FSKTmult) in three blocks, each separated by approximately thirty minutes. Again, the results indicated no significant differences in FSKTmult measures or CMJ performance between the two conditions. In conclusion, IPC did not significantly affect neuromuscular (in both studies), endurance (in the first study), or anaerobic (in the second study) performance in these taekwondo athletes.

## 1. Introduction

Taekwondo is an Olympic combat sport characterized by alternating high-intensity actions and moments of lower intensity or pauses [1,2]. The high-intensity action relies on the anaerobic pathways, whereas the oxidative system contributes to the recovery between the high-intensity actions and low-intensity actions [1,2,3,4].

In competition, victory is attained by outscoring the opponent in a ‘best of three rounds’ system, determined by the cumulative points across rounds. The athlete who wins the majority of rounds emerges as the match winner [4]. Additionally, during the competition, taekwondo athletes frequently engage in several matches in a single day [1]. This demanding schedule highlights the critical need for athletes to maintain peak physical readiness throughout the day of the competition.

Given the pivotal roles played by both the aerobic and anaerobic energy systems in taekwondo competition, it becomes imperative to assess taekwondo athletes’ performance using sport-specific tests that target these energy systems [1,2,4,5]. For instance, the Progressive Specific Taekwondo Test (PSTT) and the Multiple Frequency Speed of Kick Test (FSKTmult) have emerged as recognized and largely used taekwondo-specific tests [4,5]. Therefore, these tests can be valuable means to assess specific aspects of taekwondo performance [4,5]. Using these tests can significantly aid in gauging the effectiveness of interventions geared towards improving athletes’ capabilities, thereby contributing significantly to enhancing competition performance. It is important to emphasize that interventions should not only demonstrate feasibility but also seamlessly integrate into athletes’ routines for practical application [6]. Among the potential interventions, ischemic preconditioning (IPC) has emerged as a promising possibility [7,8,9].

IPC is a non-invasive technique, consisting of repeated bouts of muscular ischemia followed by reperfusion applied on the proximal portion of the arms or thighs [7,8,9]. The mechanisms underlying how IPC enhances sports performance are currently complex and need to be extensively investigated [8]. Although not specifically explored within the domain of taekwondo, the effects of IPC on sports performance have been studied across various sports contexts. For example, studies have indicated enhancements in anaerobic power, anaerobic capacity, and muscle strength [7,8,9]. Nevertheless, the evidence supporting the advantageous effects of IPC on performance remains inconclusive, primarily due to the heterogeneity of methodological approaches [7,9], such as the variety of IPC protocols used, way of performing the exercises, duration of the task, sports characteristic, and the level of physical conditioning of the participants [7,9], which makes it difficult to provide guidelines regarding the use of IPC in sports.

Regarding the level of physical conditioning of the participants, previous research involving IPC suggests that these types of interventions may yield greater benefits for healthy individuals compared to athletes [7]. Thus, extrapolating the results of interventions from non-athletes to athletes is a multifaceted challenge that requires careful consideration of numerous factors. A key issue is the ‘ceiling effect’ observed in athletes [10]. Due to high-performance physical levels, in general, there is a reduced potential for substantial improvements, leading to incrementally smaller enhancements in this population. Nevertheless, the usage of performance tests specific to modalities, in which athletes are well familiarized, can play a critical role. Such tests improve the accuracy of assessing an athlete’s capabilities, ensuring specificity by aligning more closely with the skills relevant to the athlete’s sport, thereby enhancing the applicability of the test results for both training and competitive scenarios. This approach can also boost athlete motivation and engagement, as athletes are generally more inclined to invest effort in tests that mirror the actual sporting environment.

The effects of IPC on the sports performance of athletes are still controversial [7,9,11]. Also, to the best of our knowledge, there is currently a lack of studies examining the effects of IPC on the performance of highly trained taekwondo athletes [7,9,11]. Therefore, two studies were conducted, focusing specifically on the assessment of endurance and anaerobic performance, which are key components in taekwondo. In the first study, the aim was to assess IPC effects on performance during a specific taekwondo endurance test [4], while the second study involved conducting three separate bouts [12], in three blocks, for a specific taekwondo anaerobic test [5]. This approach aimed to more accurately simulate the specific demands experienced during taekwondo matches and competitions that take place within a single day. Additionally, neuromuscular performance was evaluated through countermovement jumps in both studies. The present study hypothesized that IPC would have a moderate effect on endurance performance and a small effect on anaerobic performance [7,9].

## 2. Materials and Methods

The research was approved by the Universidade Federal de Minas Gerais Research Ethics Committee (approval number: 35462920.0.0000.5149), in accordance with the Declaration of Helsinki.

### 2.1. Study 1

#### 2.1.1. Participants

The ‘pwr’ package in R was used to determine the sample size for the study. Specifically, the parameters were moderate effect size (d = 0.51), as suggested by the Salvador et al. [9] study, a statistical power of 80% and a significance level set at 5% in a paired *t*-test. According to these parameters, a minimum of 25 participants would be required. Due to the nature of the sample, which comprised national and international taekwondo athletes, a significant limitation was encountered in reaching the target of 25 participants. Consequently, we proceeded with a study involving eleven subjects. While acknowledging the limitations associated with a smaller sample size, both traditional frequentist statistics were employed, including confidence intervals (which will allow for future analyses, e.g., meta-analysis) and Bayesian analysis (details of which will be provided in the statistical analysis section). The Bayesian approach appears to be a viable alternative in cases with a limited number of participants, as Bayesian statistics are not based on the central limit theorem and can yield reasonable results even with small sample sizes [13].

The athletes were recruited from the Sports Training Center (CTE) at Universidade Federal de Minas Gerais, as well as from partner teams. The inclusion criteria for this study were as follows: athletes must have a black belt in taekwondo, no lower limb injuries, a minimum of 5 years of practice, actively train for at least 8 h per week, and have competitive experience at the state, national, or international level. The exclusion criteria included sustaining any injuries during data collection, participation in weight control procedures during the study period, and involvement in competitive events during the same period.

Thus, well-trained black belt taekwondo athletes participated (4 females, 7 males; mean ± SD; age: 19 ± 3 years; body mass: 62 ± 11 kg; height: 169 ± 9 cm; practice time 7 ± 2 years; competitive level: national or international; weekly training duration: 10 ± 2 h). The athletes participated in approximately 10 training sessions per week, divided into technical–tactical and strength and conditioning training. During the study, the athletes were not in the competitive period and, as a result, did not perform any weight control procedures. Additionally, no lower limb injuries were reported. The participants were informed about the procedures and possible risks before signing the written informed consent.

#### 2.1.2. Study Design

**Figure 1:**
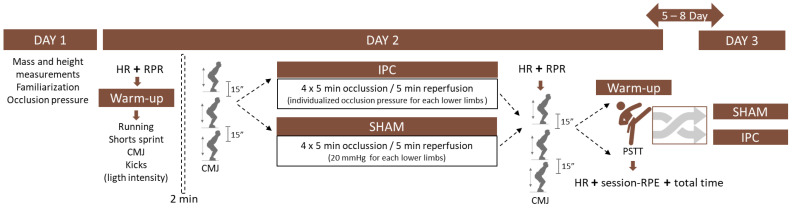
Schematic representation of the Study 1 design. Note: RPR: rating of perceived recovery HR: heart rate, CMJ: countermovement Jump, session-RPE: rating of perceived exertion of the session, PSTT: Progressive Specific Taekwondo Test, SBP: systolic blood pressure.

A randomized crossover design was utilized (Figure 1), in which each volunteer visited the laboratory on three different occasions, including one preliminary visit and two experimental visits. Randomization was performed using R software with a script that generated a random sequence. During the preliminary visit, the following steps were conducted: (1) written consent was obtained; (2) anthropometric assessment; (3) determination of occlusion pressure; and (4) familiarization with the protocols. Subsequently, each volunteer underwent two different protocols on separate occasions, with a five- to eight-day interval between them to ensure recovery from any fatigue or muscle soreness induced by the first protocol. This interval was chosen to ensure that performance during the second protocol was not adversely affected by residual fatigue. The protocols were as follows: (1) Ischemic Preconditioning Situation (IPC), when the volunteer was submitted to the IPC protocol (previously established and presented later) before the taekwondo-specific task, and (2) SHAM application, when the volunteer was submitted to the protocol that simulates the IPC (previously established and presented later) before the task. During each visit, participants reported rating of perceived recovery (RPR) and had resting heart rate (HR) and countermovement jump (CMJ) performance measured, serving as indicators of recovery status. Subsequently, participants performed a warm-up followed by either IPC or SHAM and the Progressive Specific Taekwondo Test (PSTT). At the end of the PSTT, final HR and rating of perceived exertion (RPE) were collected. The session-RPE was collected fifteen minutes after the end of the test. On the second day, the procedures of the first day were repeated but using the experimental condition not performed on the first day. All participants were instructed not to undergo any dietary restrictions and to refrain from using ergogenic substances or any other compounds that could potentially impact physical performance. Additionally, participants were advised to maintain regular eating habits before each data collection session. Furthermore, participants confirmed abstaining from engaging in intense physical activities for 24 h preceding the testing days. All visits occurred at the same time of the day, with the intention of minimizing potential interference from the diurnal variation on performance.

#### 2.1.3. Procedures

***Warm-up:*** The warm-up included one minute of self-selected light running, short-distance sprints, and countermovement jumps, followed by one minute of low-intensity taekwondo kicks performed using a kicking dummy (Boomboxe, São Paulo, Brazil).

***Countermovement Jump (CMJ):*** Two minutes following the warm-up, participants conducted three CMJs with a 15 s rest interval between each. Participants were instructed to execute maximal jumps while keeping hands on the waist and knees extended during the airborne phase. These jumps were performed using a jump mat (Elite Jump System, version 2.10, S2 Sports, São Paulo-SP, Brazil). The CMJ protocols used in this study were previously validated [14]. According to Loturco et al. [14], the criterion validity of the contact mat (Elite Jump) for measuring CMJ was very high, with an intraclass correlation coefficient of 0.997 and a very low bias of −0.08 cm, as demonstrated by the Bland–Altman plot. The average jump height across the three trials was considered for analysis [15].

***Ischemic Preconditioning Protocol (IPC):*** Initially, individualized occlusion pressure (IOP) was established for each lower limb by gradually inflating the cuff positioned in the proximal thigh area until the auscultatory pulse of the tibial artery ceased. The interruption of the pulse was verified by using a handheld Doppler (DV 610B, Medmega Indústria e Equipamentos Médicos, Franca-SP, Brazil) placed over the tibial artery in both legs [16]. Following the cessation of the pulse, value in millimeters of mercury (mmHg), an additional 20 mmHg was added to determine the IOP for each lower limb to ensure total occlusion [17].

In the IPC condition, the same cuff was positioned in the same manner as during the initial IOP determination. To induce occlusion, the cuff was inflated up to the IOP level for each lower limb. Four cycles of five minutes of occlusion (ischemia) followed by a 5 min interval of reperfusion were applied (Figure 1). For the SHAM condition, the IPC procedure was followed, although the cuff was set at 20 mmHg for all participants [18]. Throughout the application of IPC and SHAM, the primary researcher was blinded to interventions, ensuring unbiased data collection and analysis [18]. Additionally, to equalize cognitive perception of IPC and SHAM conditions, participants were informed that both conditions had the potential to improve performance [18].

***Progressive Specific Taekwondo Test (PSTT):*** The full details of the test, including its validity, have been previously provided [3,19,20]. In summary, the PSTT is a progressive taekwondo-specific test, in which athletes repeatedly perform the *bandal tchagui* technique (roundhouse kick), alternating between right and left legs. The target is a kicking dummy (Boomboxe, São Paulo, Brazil) equipped with a taekwondo body protector.

Each participant was instructed to execute the kick as in competition, aiming to score points while maintaining step movement between kicks. Verbal encouragement was provided throughout the test. The PSTT continued until voluntary exhaustion or until the athlete could not sustain the required kicking frequency or power for two consecutive kicks. Additionally, HR was continuously recorded throughout the test using an HR monitor (Polar H10, Polar Electro, Kampele, Finland). The Polar H10 sensor has previously demonstrated high reliability. McCabe et al. [21] evaluated the Polar H10 in various physical activity conditions and reported that it maintained electrode connection and transmitted heart rate data continuously, with inter-device reliability during these tests showing Pearson’s r correlation coefficients averaging 0.99.

The time to exhaustion in the PSTT served as an indirect measure of endurance performance.

### 2.2. Study 2

#### 2.2.1. Participants

As in Study 1, the ‘pwr’ package in R was used to calculate the required sample size. A small effect size (d = 0.23) was used, as suggested by Salvador et al. [9], with a statistical power of 80% and a significance level set at 5% in a paired *t*-test. According to these parameters, a minimum of 119 participants would be necessary. Therefore, Study 2 proceeded with 14 participants, and traditional frequentist statistics were performed, including confidence intervals, Bayesian analysis, and sample size calculations. The inclusion and exclusion criteria for Study 2 are the same as used in Study 1.

Fourteen black belt taekwondo athletes participated (6 females, 8 males; mean ± SD; age: 18 ± 3 years; body mass: 62 ± 13 kg; height: 169 ± 11 cm; practice time 6 ± 2 years; competitive level: national or international; weekly training time: 12 ± 2 h). Similar to Study 1, the athletes were not in the competitive period and did not perform any weight control procedures. Additionally, no lower limb injuries were reported. The participants were informed about the procedures and possible risks before signing the written informed consent.

#### 2.2.2. Study Design

**Figure 2:**
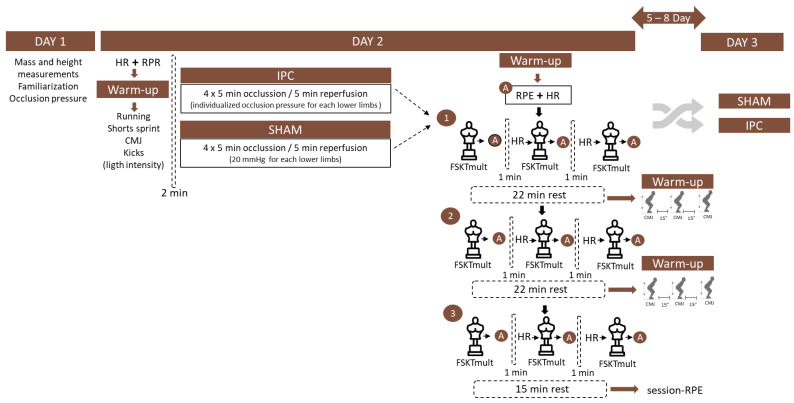
Schematic representation of the Study 2 design. Note RPR: perceived recovery status scale, HR: heart rate, CMJ: countermovement Jump, RPE: rating of perceived exertion, session-RPE: rating of perceived exertion of the session, FSKTmult: Frequency Speed of Kick test, multiple sets.

A randomized crossover design (Figure 2) was employed, where each participant visited the laboratory on three separate occasions: one preliminary visit and two experimental visits.

The procedures for Study 2 mirrored those of Study 1, including warm-up and IPC and SHAM procedures, except for the taekwondo-specific test utilized. In Study 2, the FSKTmult was implemented, consisting of three bouts with a 1 min rest interval between each bout. Additionally, three distinct blocks were structured, with thirty minutes between each block. Similar to Study 1, the CMJ, following comparable procedures, was performed between each block of the FSKTmult. On the second day, the procedures from the first day were replicated but used the alternating experimental condition (IPC or SHAM). All other protocols remained consistent with Study 1, such as participants being instructed to maintain a regular diet and refrain from engaging in vigorous physical activity 24 h before the test days.

#### 2.2.3. Procedures

The warm-up, CMJ, and IPC procedures were identical to those used in Study 1.

***Frequency Speed of Kick Test Multiple (FSKTmult):*** The FSKTmult was conducted following previous descriptions [3,22,23]. Full details regarding the validity can be found in previous studies [3,22,23,24,25]. Athletes were prompted to execute the maximum number of kicks, alternating between the right and left legs using the *bandal tchagui* technique, following a signal. The test comprised 5 sets, each lasting 10 s, with 10 s of rest between sets. It was performed on a kicking dummy (Boomboxe, São Paulo, Brazil) equipped with a taekwondo body protector.

In the present study, the FSKTmult protocol was adapted. A modified approach was employed involving three bouts of FSKTmult, with one minute of rest between each bout [12]. Additionally, three blocks separated by thirty minutes were implemented to replicate the specific demands inherent with taekwondo matches and competitions. The rating of perceived exertion (RPE) scale was also employed. Participants were required to report RPE at the conclusion of the test (final RPE). Performance was measured by the number of kicks.

#### 2.2.4. Statistical Analysis

For both studies, the distribution of each variable was examined using the Shapiro–Wilk test. Means and standard deviation were used to present the data.

Paired Student *t*-test was used to verify the difference between the conditions (SHAM and IPC). A Cohen’s d effect size was calculated [26] and interpreted using the following cut-offs: 0.1 to less than 0.3 for a small effect, 0.3 to less than 0.5 for a moderate effect, and greater than or equal to 0.5 for a large effect [27]. As previously mentioned, in cases where the requirements for the paired *t*-test were not satisfied, the Wilcoxon signed-rank test was applied. An r effect size [28] was calculated and interpreted using the following cut-offs: 0.1 to less than 0.3 for a small effect, 0.3 to less than 0.5 for a moderate effect, and greater than or equal to 0.5 for a large effect [29].

Additionally, to identify possible differences in the CMJ variable, two-way ANOVA with repeated measurements was used [2 moments (pre-intervention × post-intervention) × 2 conditions (SHAM × IPC)]. Also, Two-way ANOVA with repeated measurements was used [5 sets × 2 conditions (SHAM × IPC)]. For the effect size in the analyses using ANOVA, the partial eta squared (η_p_^2^) was used. The following scale was used to classify the effect size: trivial (>0.01), small (0.01 to 0.05), moderate (0.06 to 0.13), and large (≥0.14) [29]. The Bonferroni test was used as a post hoc test. The significance level adopted was α < 0.05.

The ‘BayesFactor’ package [30] in R was used to conduct both a Bayesian paired *t*-test and Bayesian repeated-measures analysis of variance (ANOVA). The r-scale fixed effects were set at an uninformed prior of 0.5. Bayes Factors (BF_10_) were used to provide evidence for or against the null hypothesis. The BF_10_ quantifies evidence strength, favoring the alternative hypothesis relative to the null hypothesis [31]. Values exceeding 1 and below 3 indicate “anecdotal” evidence; those from 3 up to, but not including, 10 suggest “moderate” evidence; a factor from 10 to just under 30 signifies “strong” evidence; factors between 30 and less than 100 are categorized as “very strong”; and a factor of 100 or higher represents “extreme” evidence for the alternative hypothesis. Conversely, when the BF_10_ is less than 1, the evidence supports the null hypothesis: a BF_10_ lower than 0.33 but greater than 0.1 denotes “anecdotal” support for the null; a factor from 0.1 to 0.033 indicates “moderate” evidence; a range from 0.033 to 0.01 reflects “strong” evidence; and a value less than 0.01 conveys “very strong” to “extreme” evidence in favor of the null hypothesis [31].

The statistical analysis of the data was performed using the R software, version 4.0.5.

## 3. Results

### 3.1. Study 1

#### 3.1.1. Pre-Intervention

**Figure 3:**
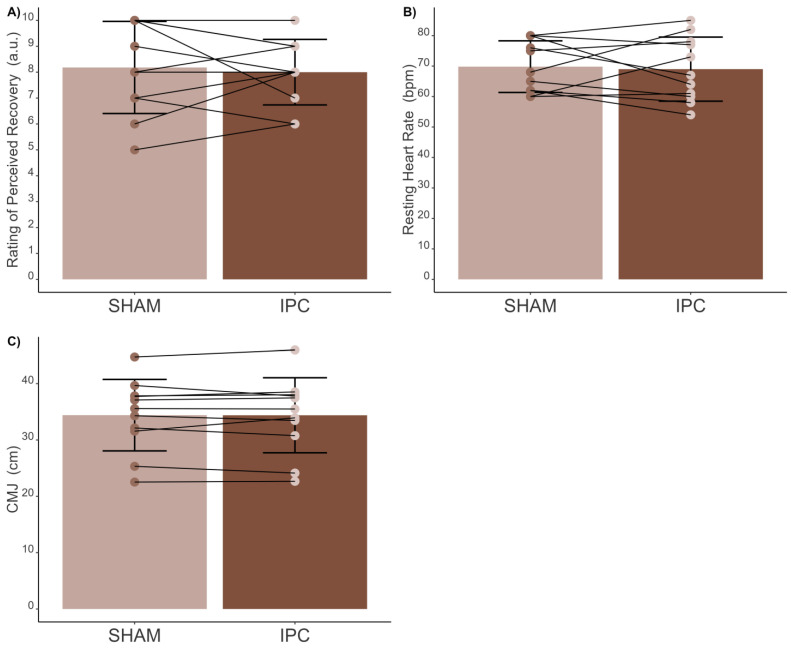
Comparison between SHAM and ischemic preconditioning (IPC) conditions before the test. (**A**) Performance Recovery Score (PRS); (**B**) resting heart rate (HR); and (**C**) countermovement jump (CMJ).

No significant differences (Figure 3A) were observed in the RPR between SHAM [8 (95% CI 7–9) ± 2 a.u.] and IPC [8 (95% CI 7–9) ± 1 a.u.] conditions [t(10) = 0.43; *p* = 0.676; d = 0.12 (trivial)], with Bayes Factor analysis indicating no evidence of differences (BF_10_: 0.32). Similarly, no significant differences in resting HR (Figure 3B) were found [t(10) = 0.30; *p* = 0.774; d = 0.086 (trivial)] between the SHAM [70 (95% CI 65–75) ± 8 bpm] and IPC [69 (95% CI 63–75) ± 11 bpm] conditions, with a Bayes Factor analysis supporting this finding (BF_10_: 0.31). Additionally, no significant differences in CMJ (Figure 3C) were observed [t(10) = −0.05; *p* = 0.958; d = 0.003 (negligible)] between the SHAM [34 (95% CI 31–38) ± 6 cm] and IPC [34 (95% CI 30–38) ± 7 cm] conditions, with the Bayes Factor analysis again indicating no evidence of differences between conditions (BF_10_: 0.30).

#### 3.1.2. Intervention

No statistically significant differences [t(10) = 1.00; *p* = 0.34] were observed in occlusion pressure for the athlete’s left [186 (95% CI 173–199) ± 22 mmHg] and right lower limbs [190 (95% CI 179–201) ± 19 mmHg].

#### 3.1.3. Post-Intervention

**Figure 4:**
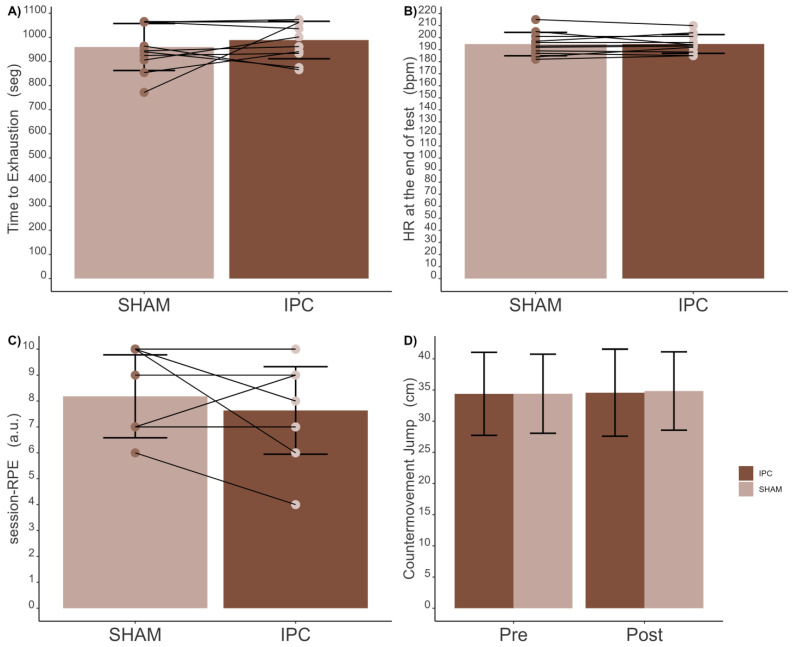
Comparison between SHAM and IPC conditions in performance-related parameters. (**A**) Time to exhaustion; (**B**) heart rate (HR) at the end of the test; (**C**) session-RPE; (**D**) countermovement jump (CMJ), pre and post.

No statistically significant differences were found in athletes’ time to exhaustion [t(10) = 0.93 (95% CI −40.53, 98.35); *p* = 0.375; d = 0.328 (small)] between the SHAM [960 (95% CI 903–1018) ± 97 s] and IPC [989 (95% CI 943–1035) ± 23 s] conditions (Figure 4A), with Bayes Factor analysis indicating no evidence of differences (BF_10_: 0.43). Likewise, no significant differences were observed in athletes’ HR at the end of the test [t(10) = 0.06 (95% CI −3.43, 3.61); *p* = 0.955; d = 0.010 (trivial)] between the SHAM [194 (95% CI 189–200) ± 9 bpm] and IPC [195 (95% CI 190–199 ± 8 bpm] conditions (Figure 4B), with a Bayes Factor analysis supporting this finding (BF_10_: 0.30). Additionally, there were no significant differences for the session-RPE (Figure 4C) [v = 6; *p* = 0.374; r = 0.273 (small)] between the SHAM [8 (95% CI 7–9 ± 2 a.u.] and IPC [8 (95% CI 7–9 ± 2 a.u.] conditions, with the Bayes Factor analysis again indicating no evidence of differences between conditions (BF_10_: 0.45).

Concerning CMJ height (Figure 4D), there were no significant effects observed for the moment [F_1;38_ = 0.013; *p* = 0.908; η_p_^2^ = 0.00027 (trivial)], the condition [F_1;38_ = 0.243; *p* = 0.625; η_p_^2^ = 0.00038 (trivial)], or the interaction between moment and condition [F_1;38_ = 0.010; *p* = 0.921; η_p_^2^ = 0.00025 (trivial)]. The pre-intervention CMJ height was 34 cm for SHAM [(95% CI: 31–38) ± 6 cm] and 34 cm for IPC [(95% CI: 30–38) ± 7 cm] conditions. Likewise, the post-intervention CMJ heights were 35 cm for SHAM [(95% CI: 31–39 cm) ± 6] and 35 cm for IPC [(95% CI: 31–39) ± 7 cm] conditions. Also, Bayes Factor analysis further supported these findings, indicating no evidence of a main effect for the moment (BF_10_: 0.39), conditions (BF_10_: 0.31), and interaction (BF_10_: 0.12).

### 3.2. Study 2

#### 3.2.1. Pre-Intervention

**Figure 5:**
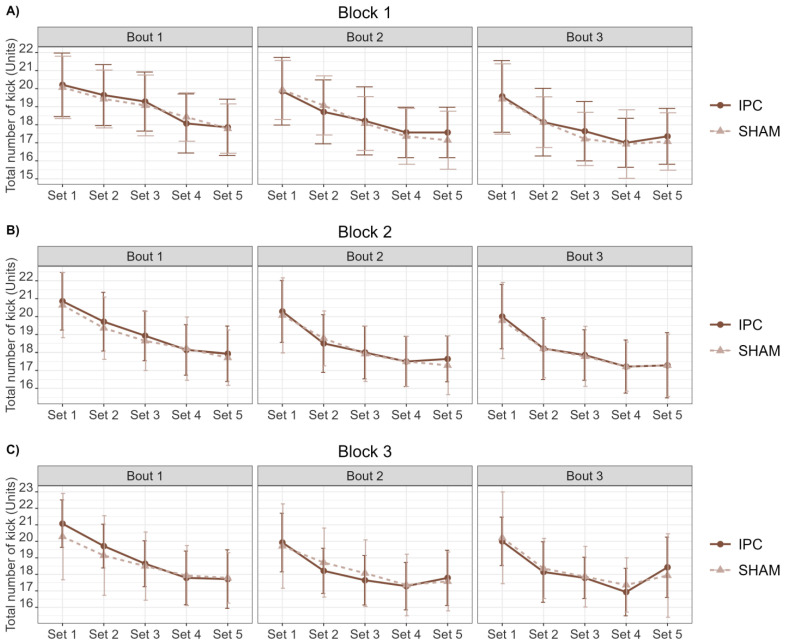
Comparison of total number of kicks between SHAM and IPC conditions across sets and bouts in three blocks. (**A**) Block 1; (**B**) Block 2; (**C**) Block 3.

There were no significant differences for the RPR (Figure 5) [v = 21; *p* = 0.901; r = 0.028 (small)] between SHAM [9 (95% CI: 8–9) ± 1 a.u.] and IPC [9 (95% CI: 8–10) ± 2 a.u.] conditions. Bayes Factor analysis indicated no evidence of differences between conditions (BF_10_: 0.35).

#### 3.2.2. Intervention

There were no significant differences [t(13) = 0.249; *p* = 0.807] for the occlusion pressure between the left [192 (95% CI: 182–202) ±18 mmHg] and right lower limbs [191 (95% CI: 181–201) ± 20 mmHg].

#### 3.2.3. Post-Intervention

Table 1 shows the results of the ANOVA with repeated measures on the total number of kicks (Figure 5) between the SHAM and IPC conditions for sets and bouts in all three blocks. Notably, a significant difference was observed only in the interaction between ‘condition’ and ‘sets’ within Block 1 during Bout 2. However, it is crucial to note that the Bonferroni post hoc analysis did not reveal any significant differences between the conditions across different ‘sets’ (*p* > 0.05), suggesting that the ‘condition’ does not have a significant effect. In the other comparisons, no significant differences were identified.

Additionally, a Bayes Factor analysis was conducted, presenting ‘Model 1’ through ‘Model 4’ as supplementary analyses. ‘Model 1’, which seems to consider only the ‘Condition’, consistently shows low BF_10_ values (around 0.18 to 0.29), suggesting weak evidence in favor of this model. ‘Model 2’, which includes ‘Set’, and ‘Model 3’, which includes both ‘Set’ and ‘Condition’, exhibit extremely high BF_10_ values, ranging from 10^12^ to 10^2^⁶, indicating strong evidence supporting these models. Moreover, ‘Model 4’, which might include the interaction (‘Set:Condition’), displayed lower higher values of BF_10_. These results suggest that ‘Set’ significantly contributes to the models, as evidenced by the lower BF10 values of ‘Models 3’ and ‘4’ when compared to ‘Model 2’, which includes only ‘Set’. This indicates that ‘Models 3’ and ‘4’ do not enhance the model’s explanatory power as effectively as ‘Model 2’.

**Figure 6:**
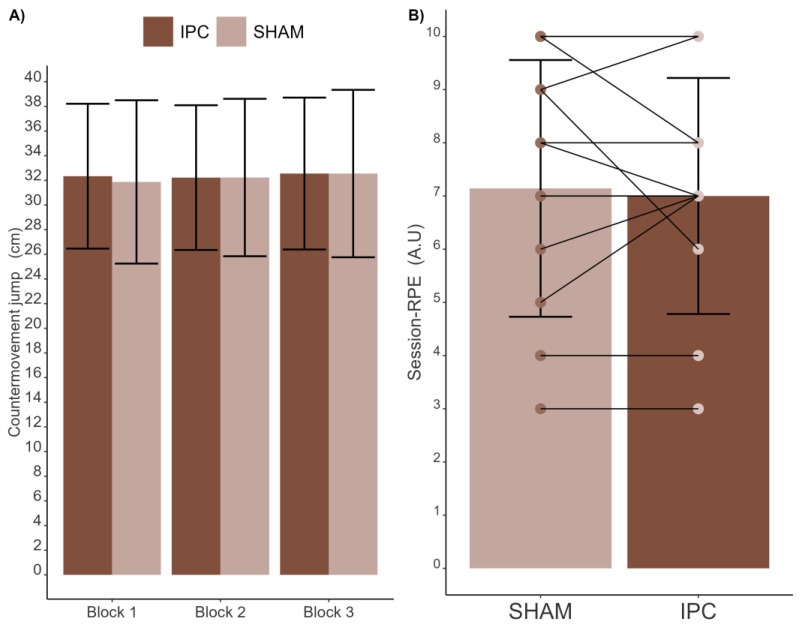
Comparison of CMJ (**A**) and RPE-session (**B**) between SHAM and IPC conditions.

Regarding CMJ (Figure 6A), there were no main effects of condition (F_1;13_ = 0.124; *p* = 0.731; η_p_^2^ = 0.00016, trivial), blocks [F_1.28;16.59_ = 0.798; *p* = 0.414; ηp^2^ = 0.00097 (trivial)], or the interaction between condition and blocks [F_2;26_ = 0.292; *p* = 0.749; ηp^2^ = 0.00033 (trivial)]. Additionally, Bayes Factor analysis further supported these findings, indicating no evidence of a main effect for the conditions (BF_10_: 0.24), blocks (BF_10_: 0.18) and interaction (BF_10_: 0.01).

No significant differences were found for the session-RPE (Figure 6B) between the SHAM [7 (95% CI: 6–9) ± 2 a.u.] and IPC (7 (95% CI: 6–8) ± 2 a.u.] conditions (t(13) = 0.434, *p* = 0.671, d = 0.116 [trivial]). Bayes Factor analysis indicated no evidence of differences between conditions (BF_10_: 0.38) in session-RPE.

## 4. Discussion

The aim of the present study was to verify the effects of IPC on the performance of taekwondo athletes. It was hypothesized that IPC would improve neuromuscular (Study 1 and 2), endurance (Study 1) and anaerobic performance in the successive blocks and bouts of a specific test (Study 2) of taekwondo athletes when compared to the SHAM condition. However, the main results of the present study showed that four cycles of five minutes of occlusion of bilateral blood flow of the lower limbs with occlusion pressure defined individually, interspersed with five minutes of reperfusion, did not influence any of the variables assessed.

A study conducted by Campos et al. [32] estimated that during simulated taekwondo matches, athletes primarily rely on the oxidative system (62–74%), with a lower reliance on anaerobic systems (ATP-PCr: 19–33%; glycolytic: 3–9%). Additionally, Apollaro et al. [4] highlighted that evaluating endurance performance in taekwondo athletes using generic physical exercise, such as treadmills or cycle ergometers, could be less consistent than specific tests. Therefore, multiple studies have emphasized the necessity of employing specific tests for assessing endurance performance [1,4,5]. In the first study, the hypothesis of the beneficial effects of IPC on endurance performance and CMJ in taekwondo athletes was based on previous studies that found positive effects on the time to exhaustion [33,34] and on maximal oxygen consumption [33,35]. Additionally, the hypothesis considered findings from previous reviews [7,8,11], which explored the underlying mechanisms of IPC contributing to decreased blood lactate levels during incremental exercise, enhanced efficiency in muscular oxygen utilization, and the moderation of the typical hypoxic increase in pulmonary artery pressures. Although the physiological underpinnings of IPC’s effects are still somewhat speculative, it is essential that future studies investigate and characterize the molecular and biological mechanisms driving IPC’s effects [7]. However, despite existing studies for the potential benefits of IPC for physical performance, the present study did not demonstrate any significant effect of IPC on the endurance performance of taekwondo athletes during specific tests.

In both studies, the indirect lower limb muscular power of taekwondo athletes was assessed using the CMJ, which is the most commonly used test by taekwondo athletes [1,3]. Muscle power appears to be a crucial factor in taekwondo performance. For instance, Goulart et al. [36] showed a positive correlation between CMJ and the speed of the *bandal tchagui* kick. Similarly, Albuquerque et al. [3] identified a moderate positive correlation between CMJ and the total number of kicks in one bout of the FSKTmult test. Thus, enhancing CMJ performance through IPC could potentially have a meaningful impact on taekwondo performance. Nonetheless, the results did not demonstrate any IPC-related benefits in CMJ performance. This lack of observed benefit may be attributed to the timing that IPC was performed in relation to when the performance tests were administered. For example, a study by Beaven et al. [37] examined the impact of IPC on CMJ performance across two distinct timeframes: immediately following the intervention and 24 h later. A significant positive effect of IPC on certain CMJ metrics was observed in measurements taken 24 h post-intervention but not immediately afterwards. The authors hypothesized that hypoxia may impair muscle spindle responsiveness, leading to alterations in sensorimotor control [37]. Additionally, it was posited that discomfort resulting from the occlusive pressure during IPC could be a factor in mitigating the possible immediate effects of IPC on CMJ performance [37].

Similarly, a study conducted by Ceylan and Franchini [38] with elite judo athletes suggests a potential ‘delayed’ benefit of IPC. The study revealed that while IPC does not immediately enhance performance in judo-specific tests among elite judo athletes, a finding consistent with observations in taekwondo, it does contribute to improved recovery. Further, Ceylan et al. [39] demonstrated that IPC, when applied to judo athletes after judo-specific exercises, enhanced the recovery measures.

In the second study, the widely recognized and extensively used taekwondo-specific test, FSKTmult, was employed [3,22,23,40]. This test is known for its reliability, sensitivity, simplicity, and cost-effectiveness that replicates key aspects of taekwondo, primarily focusing on assessing anaerobic fitness [23]. However, to better replicate the specific demands inherent in taekwondo matches and competitions, an approach involving three bouts of FSKTmult, with one minute of rest between each bout, was employed [12]. Furthermore, the study was organized into three blocks, each separated by thirty minutes. This design was deliberately chosen to closely mirror the specific demands in taekwondo matches and competitions. It is important to emphasize that this aspect of the study design enhances the applicability and relevance of the findings to taekwondo. Also, this procedure allowed us to examine the immediate to delayed effects of IPC by introducing multiple bouts interspersed with 30 min intervals. It was expected that if IPC enhances recovery more effectively than a SHAM condition, this would lead to better performance in the later bouts of the third block. On the other hand, the present study did not find evidence to support this possible effect of IPC in taekwondo performance.

In addition, the hypothesis regarding the advantageous effects of IPC on multiple bouts and blocks of FSKTmult is derived from psychophysiological effects. These include bidirectional body–brain integration and a spectrum of hemodynamic responses [8]. Such responses involve heart vagal modulation, vasodilation, the release of humoral factors, and increased muscle oxygen extraction. Consequently, the possible psychophysiological alterations induced by IPC could potentially enhance multiple bouts and blocks of FSKTmult performance by delaying exhaustion onset, modifying perceptions, and inducing behavioral changes [8]. However, the results of the present study indicated that IPC did not induce changes in anaerobic performance responses in successive bouts and blocks of FSKTmult.

A critical aspect to consider in this study, as with any research involving IPC, is the protocol used. Currently, there is no consensus on the optimal procedure for an IPC intervention [7,11]. Generally, reviews have shown that most IPC studies used a randomized crossover design [7,11]. The number of ischemia and reperfusion cycles ranged from two to eight, and the duration of occlusion periods fluctuated between two and ten minutes [7]. Additionally, most studies performed IPC on the same day as the test [7]. The time interval between the administration of the IPC protocol and the commencement of the tests also differs widely, ranging from immediate to delayed periods (e.g., up to 72 h) [7]. In the present study, a randomized crossover design was employed, wherein participants underwent four cycles of occlusion, each lasting five minutes, followed by five-minute intervals of reperfusion. This approach was based on previous studies, such as Cocking et al. [41], which indicated that traditional IPC [4 × 5 min thigh (bilateral)] provided more benefits to exercise performance compared to SHAM conditions. Additionally, Cocking et al.’s [41] study suggested no added advantage in increasing the number of cycles or using unilateral IPC.

Another important methodological issue is the determination of the arterial occlusion pressure. In general, some researchers who used IPC employed a pressure cuff inflated to 200 or 220 mmHg for all participants [7,11], regardless of individual differences. A practical application of IPC without considering individual differences can impact the discomfort of the method [11]. This lack of customization may lead to unnecessary discomfort or even pain, particularly for those not accustomed to high levels of pressure. Furthermore, excessive pressure could potentially alter the psychophysiological responses intended by the IPC. Therefore, considering each participant’s tolerance and adjusting the occlusion pressure accordingly could not only reduce discomfort but also ensure more accurate and reliable outcomes in IPC studies. Also, as demonstrated by Laurentino et al. [16], the handheld Doppler provides an affordable and valid method for determining resting arterial occlusion pressure, offering a safer and more relative stimulus for athletes during IPC. In the present study, the individual occlusion pressure was established for each lower limb by gradually inflating the cuff placed on the proximal thigh area until the auscultatory pulse of the tibial artery ceased. This interruption was verified using a handheld Doppler placed over the tibial artery in both legs. Therefore, it is assumed that the individualized approach ensured effective occlusion to elicit the desired physiological responses and benefits, while also minimizing potential discomfort for the participants.

A key limitation of the present study was related to the small sample sizes in both studies. An a priori sample size calculation analysis suggested a need for at least 25 participants in the first study and 119 in the second study. However, recruiting such numbers proved challenging [38,39], due to the specific cohort of the present sample, which consisted of national and international taekwondo athletes. It is crucial to emphasize the level of the athletes that participated in this study. The sample consisted of both current and former members of the Brazilian Junior and Adult Taekwondo National Teams. These athletes possess considerable experience in international competitions, including the Junior and Adult World Championships. Additionally, all participants in both studies had experience in prestigious national competitions, such as the Brazilian Taekwondo Grand Slam and Brazilian Taekwondo Championship.

Therefore, while acknowledging the limitations associated with a smaller sample size, publishing the results is deemed essential to enhance knowledge on this topic. To address the limitation of a small sample size, both traditional frequentist statistics, including confidence intervals, and Bayesian analysis were used. This approach not only enhances the robustness of the findings but also facilitates future research, such as meta-analysis. In addition, Bayesian analysis offers an alternative perspective in situations with a limited number of participants. Unlike frequentist methods, Bayesian statistics are not based on the central limit theorem, making this particularly suitable for analyses with smaller sample sizes [13].

Moreover, extrapolating intervention results from non-athletes to athletes presents a complex challenge, necessitating the consideration of various factors, including in IPC studies [7]. First, athletes typically display limited scope for performance enhancement, a phenomenon that may be partly attributable to the ‘ceiling effect’ [10]. This effect emerges due to years of training, positioning these individuals near physical capabilities, where noticeable improvements become increasingly marginal [10]. Furthermore, accurately gauging performance enhancements in elite athletes is notably arduous. The subtlety of these improvements demands highly sensitive assessment techniques capable of detecting minute changes. Therefore, it is possible to speculate that the taekwondo assessments used in the present study may not be sufficiently sensitive to detect subtle variations within this specific group of participants [42]. For instance, consider a hypothetical scenario where two athletes each completed 18 kicks in the first set of the first block of the FSKTmult. However, while the first athlete finished the 18th kick just before the end of the 10 s, the second was on the verge of executing the 19th kick at the same point [42]. This scenario suggests that a more precise method of measurement could enhance the test’s ability to identify minor changes. Therefore, the test’s sensitivity to small changes is particularly vital in the assessment of athletes, where even minor performance differences can be crucial. Thus, new studies with more “sensitivity” measures to identify changes in taekwondo athletes need to be considered.

Future research is particularly important, including the exploration of various IPC protocols, focusing on different occlusion and reperfusion durations, and expanding the sample size is essential. Additionally, maintaining specific tests while adopting more sensitive measures for future studies is imperative. Furthermore, incorporating physiological measures, such as lactate concentrations, and examining the psychological influences of IPC could yield a more comprehensive understanding of IPC.

## 5. Conclusions

Ischemic preconditioning (IPC) has garnered interest as a potential intervention to enhance sports performance. However, the present study shows that IPC, consisting of four cycles of occlusion (ischemia) using individualized occlusion pressure lasting five minutes each, followed by a 5 min interval of reperfusion, does not significantly impact the endurance and anaerobic performance of taekwondo athletes.

## 6. Practical Applications

Ischemic preconditioning (IPC) has attracted interest as a potential method to improve sports performance. However, the present study shows that IPC does not significantly impact taekwondo athletes’ performance, as determined by commonly used endurance and anaerobic tests specific to taekwondo. This suggests that IPC might not be an effective strategy for enhancing performance in taekwondo. Therefore, coaches and athletes should exercise caution when considering the incorporation of IPC into training and competition routines.

## Figures and Tables

**Table 1:** Repeated-measures ANOVA and Bayes Factor results of ‘Conditions’ (SHAM and IPC), sets, and interactions in all blocks and bouts of FSKTmult.

		Repeated Measure ANOVA			Bayes Factor Analysis			
		Condition	Set	Interaction	Model 1	Model 2	Model 3	Model 4
Block 1	Bout 1	F_1;13_ = 0.055; *p* = 0.818 η_p_^2^ = 0.004	F_2.05;26.71_ = 68.192; *p* < 0.001 η_p_^2^ = 0.840	F_4;52_ = 1.677; *p* = 0.169 η_p_^2^ = 0.114	BF_10_: 0.22	BF_10_: 6.29 × 10^21^	BF_10_: 1.23 × 10^21^	1.90 × 10^20^
	Bout 2	F_1;13_ = 0.079; *p* = 0.784 η_p_^2^ = 0.006	F_2.56;33.34_ = 89.136; *p* < 0.001 η_p_^2^ = 0.873	F_4;52_ = 2.642; *p* = 0.044 η_p_^2^ = 0.169	BF_10_: 0.19	BF_10_: 6.60 × 10^21^	BF_10_: 1.36 × 10^21^	3.40 × 10^20^
	Bout 3	F_1;13_ = 0.657; *p* = 0.432 η_p_^2^ = 0.048	F_2.32;30.18_ = 56.041; *p* < 0.001 η_p_^2^ = 0.812	F_4;52_ = 0.457; *p* = 0.767 η_p_^2^ = 0.034	BF_10_: 0.25	BF_10_: 2.67 × 10^20^	BF_10_: 1.14 × 10^20^	9.92 × 10^18^
Block 2	Bout 1	F_1;13_ = 0.978; *p* = 0.341 η_p_^2^ = 0.070	F_1.52;19.71_ = 54.030; *p* < 0.001 η_p_^2^ = 0.806	F_4;52_ = 0.858; *p* = 0.495 η_p_^2^ = 0.062	BF_10_: 0.25	BF_10_: 4.72 × 10^26^	BF_10_: 2.59 × 10^26^	2.22 × 10^25^
	Bout 2	F_1;13_ = 0.127; *p* = 0.728 η_p_^2^ = 0.010	F_1.75;22.76_ = 39.205; *p* < 0.001 η_p_^2^ = 0.751	F_2.34;30.40_ = 1.163; *p* = 0.332 η_p_^2^ = 0.082	BF_10_: 0.18	BF_10_: 6.32 × 10^21^	BF_10_: 1.22 × 10^21^	1.51 × 10^20^
	Bout 3	F_1;13_ = 0.054; *p* = 0.820 η_p_^2^ = 0.004	F_1.90;24.76_ = 32.660; *p* < 0.001 η_p_^2^ = 0.715	F_1.91;24.85_ = 0.127; *p* = 0.873 η_p_^2^ = 0.010	BF_10_: 0.19	BF_10_: 3.85 × 10^17^	BF_10_: 7.46 × 10^16^	4.70 × 10^15^
Block 3	Bout 1	F_1;13_ = 0.843; *p* = 0.375 η_p_^2^ = 0.061	F_1.84;23.94_ = 61.972; *p* < 0.001 η_p_^2^ = 0.827	F_1.98;25.80_ = 0.797; *p* = 0.186 η_p_^2^ = 0.121	BF_10_: 0.29	BF_10_: 1.62 × 10^20^	BF_10_: 9.21 × 10^19^	2.60 × 10^28^
	Bout 2	F_1;13_ = 0.185; *p* = 0.674 η_p_^2^ = 0.014	F_1.98;25.76_ = 21.387; *p* < 0.001 η_p_^2^ = 0.622	F_4;52_ = 1.830; *p* = 0.137 η_p_^2^ = 0.123	BF_10_: 0.19	BF_10_: 6.33 × 10^12^	BF_10_: 1.36 × 10^12^	2.08 × 10^11^
	Bout 3	F_1;13_ = 0.079; *p* = 0.783 η_p_^2^ = 0.006	F_2.34;30.38_ = 23.807; *p* < 0.001 η_p_^2^ = 0.647	F_4;52_ = 0.1.243; *p* = 0.304 η_p_^2^ = 0.087	BF_10_: 0.19	BF_10_: 7.25 × 10^12^	BF_10_: 1.38 × 10^12^	1.84 × 10^11^

Note: ‘Model 1’—Condition; ‘Model 2’—Set; ‘Model 3—Condition and Set; ‘Model 4’—Condition: Set (interaction).

## Data Availability

The data that support the findings of this study are available from the corresponding author (M.R.A.) upon reasonable request.
